# Medicinal Use of Wine

**Published:** 1855-02

**Authors:** Bence Jones


Medicinal use of Wine.—As a diuretic, Moselle may be as useful as gin or whiskey. In contains an excess of salts, and may prove as energetic as, and less heating than, the essential oil in gin or whiskey.

In diabetes, use the claret wines, which are free from sugar, and contain much tannic acid.

In dyspepsia and gout, use the wine most free from ultimate acidity and least stimulating. The best is the least acid claret wine; next, strong or perfectly dry champagne. Good Mansanilla will answer the purpose, and is much cheaper than Amontillado sherry. But, after all, weak brandy and water, or some pure spirits and water, is most free from acid and sugar, and answers best in dyspepsia.—*Dr. Bence Jones.*

